# Expression of *Pinellia pedatisecta* Agglutinin *PPA* Gene in Transgenic Sugarcane Led to Stomata Patterning Change and Resistance to Sugarcane Woolly Aphid, *Ceratovacuna lanigera* Zehntner

**DOI:** 10.3390/ijms23137195

**Published:** 2022-06-28

**Authors:** Mengyu Zhao, Yuming Zhou, Liangyinan Su, Guomeng Li, Zizhou Huang, Dunyou Huang, Weimin Wu, Yang Zhao

**Affiliations:** 1 State Key Laboratory of Conservation and Utilization of Subtropical Agro−Bioresources, Guangxi University, Nanning 530004, China; 2017391041@st.gxu.edu.cn (M.Z.); 18871780890@163.com (Y.Z.); 2017301033@st.gxu.edu.cn (L.S.); 2017301025@st.gxu.edu.cn (G.L.); 1831200408@st.gxu.edu.cn (Z.H.); 1831200305@st.gxu.edu.cn (D.H.); 1831201221@st.gxu.edu.cn (W.W.); 2 Guangxi Key Laboratory of Sugarcane Biology, Guangxi University, Nanning 530004, China

**Keywords:** sugarcane, *PPA*, lectin, sugarcane woolly aphid, aphid resistance

## Abstract

The sugarcane woolly aphid is one of the main pests of sugarcane worldwide. The *Pinellia pedatisecta agglutinin* (*PPA*) gene has been demonstrated to function towards aphid resistance in other crops. In our study, in order to investigate the *PPA* function towards aphid control in sugarcane and its underlying mechanism, the *PPA* gene was overexpressed in a sugarcane Zhongzhe 1 (ZZ1) cultivar in independent transgenic sugarcane lines. It was confirmed in this study that *PPA* transgenic sugarcane can resist aphids via detecting the aphids’ development and tracing the survival number on *PPA*−transgenic sugarcane lines as well as *PPA* negative control lines. The mechanism of *PPA* lectin−associated defense against aphids was preliminarily explored. Stomatal patterning differences of sugarcane leaves between *PPA*−transgenic sugarcane lines and negative control lines were found. *PPA* overexpression led to an increase in stomata number and a decrease in stomata size that might have changed the transpiration status, which is critical for aphids’ passive feeding. Moreover, the antioxidant enzyme, sugar, tannin and chlorophyll content in sugarcane leaves before and after aphid infestation was determined. The results indicated that *PPA* overexpression in sugarcane resulted in an increase in antioxidant enzyme activity and tannin content, as well as a reduction in the decline of certain sugars. These together may improve sugarcane resistance against the sugarcane woolly aphid.

## 1. Introduction

Pests are not only the most harmful organisms affecting crop yield loss and important economic factors, but are also the vectors of some plant diseases and viruses, which seriously threaten food safety and have caused huge economic loss. At present, there are two main anti−insect methods. One approach is chemical control through the application of pesticides, which can cause unavoidable side effects, such as pesticide residues, environmental pollution, incomplete insect resistance, and so on. The second approach is crop breeding to develop insect−resistant cultivars, mainly via genetic engineering. *Bacillus thuringiensis* (Bt) crystal protein genes have been widely used in crops to resist chewing pests [1,2]. Nevertheless, transgenic *Bt* crops can resist only limited types of pests. In addition, long−term *Bt* screening would lead to the evolution of pest populations and weaken the resistance effect of Bt proteins [3]. Therefore, more candidate genes that have a potential insect resistance function are needed for crop molecular improvement.

Lectins were first discovered in 1888 by Stillmark in the castor bean plant (*Ricinus communis* L.) [4] and later detected in the seeds, roots, stems, leaves and other plant tissues of a wide variety of plants. A new classification system was established for the lectin superfamily in higher plants, which mainly includes twelve families [5]. Phytolectin is a kind of protein with certain coagulation activity that can recognize and reversibly bind monosaccharides or oligosaccharides [6]. Its functions are to agglutinate cells and precipitate glycoproteins [7,8]. Functional studies have proved that monocotyledonous mannose−binding lectins have a toxic effect on stinging insects, such as lepidoptera, homoptera and brown planthoptera, especially on aphids, planthoptera and lepidoptera [9,10,11].

Several kinds of phytolectins have been studied and applied in crop improvement. *Galanthus Nivalis agglutinin* (*GNA*) was one of the earliest lectins discovered and most studied. It showed an obvious anti−insect effect in *GNA* transgenic crops such as rice, wheat, maize and cotton [12,13,14,15]. Additionally, numbers of the aphid Myzus persicae (*Sulzer*) upon inoculated transgenic tobacco plants expressing Pinellia ternate agglutinin (*PTA*) decreased by 89~92%, while the larval survival number of whitefly (*Bemisia tabaci*) decreased by 91~93% [11]. Furthermore, the expression of the Monstera deliciosaagglutinin (*MDA*) gene in tobacco enhanced plant resistance to peach−potato aphids [16].

*Pinellia pedatisecta Agglutinin* (*PPA*) is a kind of plant protein isolated from tubers of Pinellia pedatisecta belonging to the monocotyledon mannose−binding lectins, which can reversibly bind mannose or mannan, exclusively [17]. Studies have found that *PPA* from the tubers of Pinellia palmata that was introduced into cotton and peach had obvious lethal effect on aphids [9]. After that, the *PPA* gene was also transformed into tobacco and wheat, which were conferred obvious resistance to aphids [18,19]. Therefore, the *PPA* gene was considered to be a candidate gene for insect resistance, particularly against aphids.

The sugarcane woolly aphid, Ceratovacuna lanigera Zehntner (Hemiptera: Aphididae) is a widespread major pest of sugarcane. It was first reported in West Bengal in 1958, and has spread widely, with an outbreak in 2004 [20,21]. The nymphs and adults of the aphid remain on the underside of leaves and suck the sap, converting excess sugar into an excretion called “honeydew”. Honeydew secretion can cause the growth of the sooty mold fungus, which can lead to 20% or more economic loss.

The aphid−resistant sugarcane varieties showed significant differences in stomatal morphology compared to aphid−susceptible sugarcane [22]. In addition, it has been shown that the presence of tannins not only reduces the appetite of insects, but also affects their digestive systems; thus, plants with a high tannin content are more resistant to insects [23]. As far as aphids are concerned, both *Acyrthosiphon pisum* and *Myzus persicae* have been found to obtain large amounts of sugar from their host plants, and sugars, including fructose and sucrose, are major components of the “honeydew” aphid excretion. However, there are no studies revealing the interaction between sugarcane and aphids, or the mechanisms with which resistant sugarcane varieties resist aphids. We would also like to understand whether the PPA−mediated aphid resistance shared an underlying mechanism similar to that of the natural sugarcane varieties.

In this study, in order to explore whether *PPA* can be applied in sugarcane for aphid control and to study the underlying mechanism, the *PPA* gene was successfully overexpressed in the sugarcane variety ZZ1. A qRT−PCR expression analysis and a Ceratovacuna lanigera Zehntner resistance analysis were performed on independent transgenic sugarcane lines to verify the aphid resistance effect of the *PPA* gene in sugarcane. Additionally, to explore the mechanism of aphid resistance in *PPA*−transformed plants, stomata patterning was detected, and antioxidant enzyme activity and the chlorophyll, sugar and tannin content of the leaves before and after insect infestation were measured. The results of the study showed that the transformation of the *PPA* gene improved the resistance of sugarcane to the sugarcane woolly aphid, and influenced the insect resistance ability of sugarcane in terms of plant physiological and biochemical characteristics.

## 2. Results

### 2.1. PPA Transgenic Sugarcane Lines Were Obtained, and Expression Levels of the PPA Gene Were Analyzed

**Figure 1:**
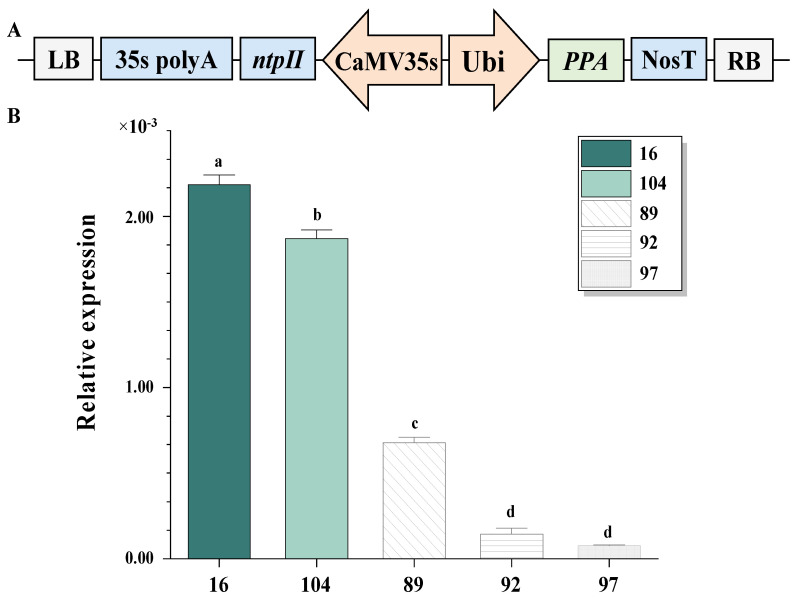
Transformation vector and expression analysis of *PPA* gene in sugarcane. (**A**) Schematic structure of the recombinant pCAMBIA2301—*PPA* vector. (**B**) Relative expression of *PPA* gene in transgenic sugarcane lines and negative control lines. Samples 16 and 104 were *PPA* transgenic sugarcane lines and the other control plants were negative lines from the same group of transformations. Each value was the mean (±SE) of three replicates, and the letters (a–d) in the figures indicated a significant difference between transgenic sugarcane lines and the control lines (one–way ANOVA, *p* < 0.05).

The *PPA* gene was subcloned into a pCAMBIA2301 vector driven by a maize ubiquitin promoter (Ubi). The selectable marker gene neomycin *phosphotransferaseII* (*nptII*) was driven by the CaMV 35S promoter (Figure 1A). Double digestion experiments were performed to verify the constructed vector, which was bombarded into sugarcane Zhongzhe 1 (ZZ1) direct embryos. After selection and regeneration, two *PPA* transgenic sugarcane lines, PPA−16 and PPA−104, were successfully obtained with a transformation efficiency of 2.0%. The transgenic positive lines were confirmed via PCR by amplifying a 150 bp region product, as expected, using the primers ppa−F (5′−ATGGCCTCCAAGCTCCTCC−3′) and ppa−R (5′−GACCAAGTCGAAGTCGCCG−3′). The relative expression of the *PPA* gene in transgenic sugarcanes was significantly higher than in the *PPA*−negative control plants, 89, 92 and 97 lines from the same group of transformation processes (Figure 1B).

### 2.2. Overexpressing PPA in Sugarcane Improves the Resistance of Sugarcane to Aphids

**Figure 2:**
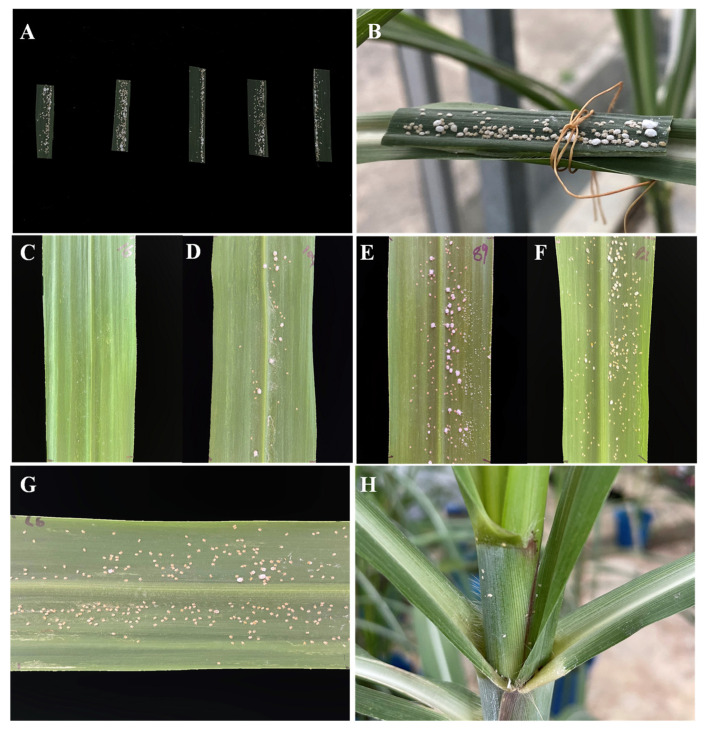
Aphid resistance tests. (**A**) Preparation of the leaf sections with equally distributed sugarcane woolly aphids. From left to right, PPA−16, 89, 92, 97 and PPA−104 plants were inoculated with leaf sections. (**B**) Leaf sections with aphids were gently tied to the positive fourth leaves of sugarcane lines. Aphid status was recorded daily, and conditions after five days are shown in (**C**) PPA−16; (**D**) PPA−104; (**E**) 89; (**F**) 92; (**G**,**H**) 97.

Two transgenic sugarcane lines PPA−16 and PPA−104 with three control plants were used to verify their resistance to aphids. Leaf sections with equally distributed sugarcane woolly aphids were tied onto the positive fourth leaves of PPA−16, 89, 92, 97 and PPA−104 plants for three replications. The status of the aphids was recorded daily for five days (Figure 2). The temperature and humidity of the greenhouse were controlled and recorded every day to avoid the influence of the external environment. The temperature of the greenhouse was never higher than 35 °C and the humidity was maintained at 50%, which was a suitable environment for the growth of aphids.

**Figure 3:**
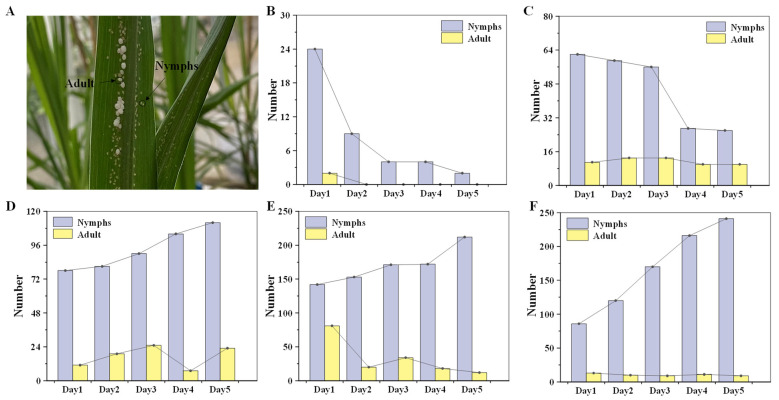
Aphid analysis. (**A**) Method of distinguishing between nymphs and adult aphids: the white individuals surrounded by honeydew were adults and the yellow individuals with less honeydew secretion were nymphs. Numbers of aphid nymphs and adults for five days after inoculation are shown in (**B**) PPA−16; (**C**) PPA−104; (**D**) 89; (**E**) 92; (**F**) 97.

**Figure 4:**
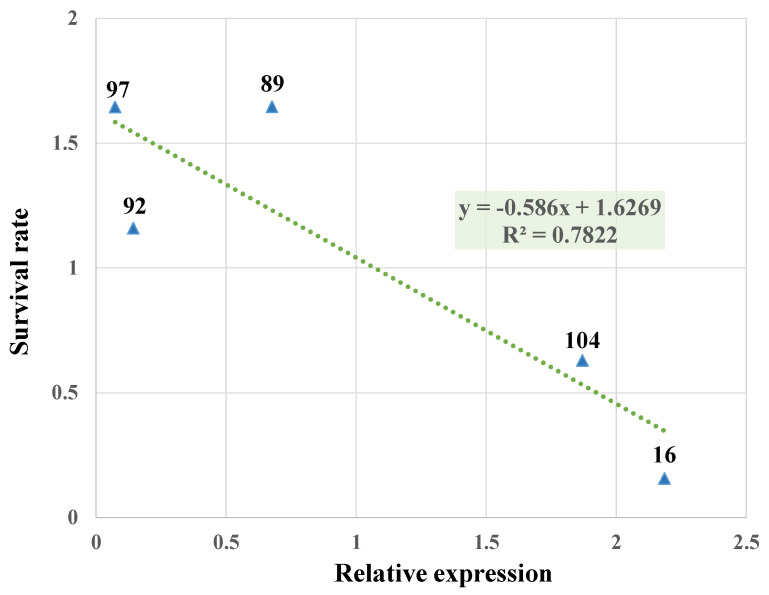
Linear regression equation. The linear regression equation was calculated using the relative expression of the PPA gene as the independent variable and the survival rate of aphids after five days as the dependent variable. Each value is the average of three replicates of the relative expression and aphid survival rate of the samples.

As shown in Figure 2, the number of aphids introduced onto the transgenic sugarcane PPA−16 were almost completely reduced to death. Only one nymph survived on the second day of inoculation. The number of nymphs on PPA−104 decreased during the first three days but showed a clear trend of mortality on day four, while the number of adults on PPA−104 constantly fluctuated but decreased overall. Aphids on the control sugarcane lines gradually increased. After five days, only the white secretion of the aphids called honeydew was left on the leaves of the PPA−positive plants PPA−16, whereas the aphids had all died (Figure 3A−C). Contrarily, aphids were present and developed well on the control plants, especially line 97 (Figure 3D−F). On line 97, aphids had even invaded the whole plant (Figure 3F), moving to stems and other leaves. Subsequently, we inoculated the plants again with twice the number of aphids, and the results were similar to previous experiments (Supplementary Figures S1−S5); thus, *PPA* transgenic lines have relatively stable aphid resistance. According to the results of the three insect feeding experiments, the linear regression equation (y = −0.586x + 1.6269, R^2^ = 0.7822) between the expression of the PPA gene and the survival rate of aphids was calculated, which indicated that *PPA* gene expression and aphid survival are negatively correlated (Figure 4).

### 2.3. Stomata Patterning Changed: Smaller and More Stomata Appeared on the Back of PPA Transgenic Sugarcane Leaves

**Figure 5:**
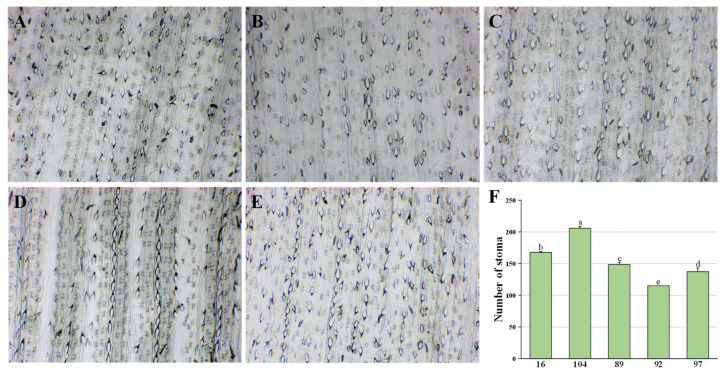
Microscopic observation of the stomata on sugarcane leaves in transgenic and control sugarcane lines. (**A**) 89; (**B**) 92; (**C**) 97, stomata on back of control sugarcane leaves observed by optical microscope with magnification 300X; (**D**) PPA−104; (**E**) PPA−16, stomata on back of transgenic sugarcane leaves observed by optical microscope with magnification 300X; (**F**) number of stomata on back leaves for each 1.285 mm^2^ area. All data are presented as the mean of three replicates with standard error (SE). The letters (a–e) in the figures indicate a significant difference among stomata of experimental sugarcanes (one−way ANOVA, *p* < 0.05).

Stomata have been reported to be associated with aphids feeding. Stomata closure can be triggered by aphids to maintain the osmotic pressure for aphid passive feeding [24]. Thus, in this study, we examined the leaf stomata patterning of the *PPA* transgenic sugarcane lines and the control lines. Abaxial stomata on sugarcane leaves can be clearly observed at 300 times magnification after debugging. The stomata distribution on the back surface of sugarcane leaves with a magnification of 300 X was determined for three replications and is shown in Figure 5. The stomata of transgenic sugarcanes PPA−16 and PPA−104 were identified as being smaller than those of the control sugarcane lines. In addition, the stomata of PPA−16 and PPA−104 increased in number counted by each 1.285 mm^2^ and were densely arranged in two or three rows. Contrarily, the stomata of the control sugarcanes were arranged loosely only in single or double rows. PPA−104 showed the highest number of abaxial stomata on back leaves, followed by PPA−16 and finally by the control sugarcanes. This indicated that stomata patterning was changed by the overexpression of *PPA* and might have influenced plant transpiration and changed the osmotic pressure, which would not be beneficial to aphids passive feeding from the xylem.

### 2.4. PPA Transgenic Lines Showed Higher Antioxidant Enzyme Content after Infestation

**Figure 6:**
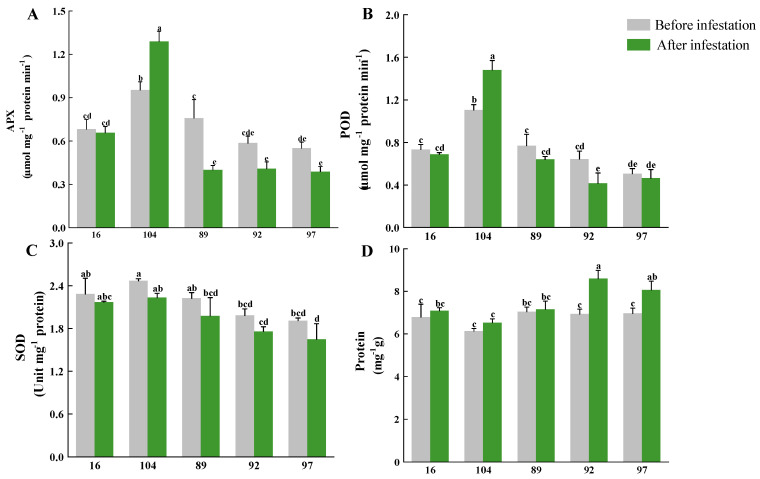
Effects of *PPA* overexpression on the activity of antioxidant enzymes and content of total proteins under infestation. Activities of (**A**) APX; (**B**) POD; (**C**) SOD and content of (**D**) proteins in leaves before and after infestation were shown; all data were presented as the mean of four replicates with standard error (SE). The letters (a–e) in the figures indicate a significant difference among experimental sugarcane lines (one−way ANOVA, *p* < 0.05).

Three antioxidant enzymes and total proteins were measured in the plant leaves before and after aphid infestation. Apparently, there was no significant difference in the content of the three antioxidant enzymes between the *PPA* transgenic lines and control lines before infestation, indicating that physiologically, the transformation of the *PPA* gene did not affect the antioxidant system of the plants without pest infestation. After the aphid infestation, all the antioxidant enzymes of the control lines dropped in content as expected, and were all lower than that of the *PPA* transgenic lines. The content of APX and POD both significantly varied between transgenic and control lines after infestation (Figure 6A,B), whereas SOD did not (Figure 6C). The APX and POD content of transgenic sugarcane PPA−104 increased significantly after infestation, and slightly declined in PPA−16. The SOD content of PPA−16 and PPA−104 both dropped, but the decline was lower than that of the control lines. The antioxidant enzymes function as protective factors under conditions of adversity. A higher antioxidant enzyme content gave plants an enhanced resistance to adversity. In addition, the total amount of proteins increased in all with no significant difference detected. This indicates that the infestation triggered protein upregulation for both *PPA* transgenic and control lines.

### 2.5. Sugar Content Dropped after Aphid Infestation: PPA Transgenic Lines Exhibited Less Decline in Soluble Sugar and Sucrose

**Figure 7:**
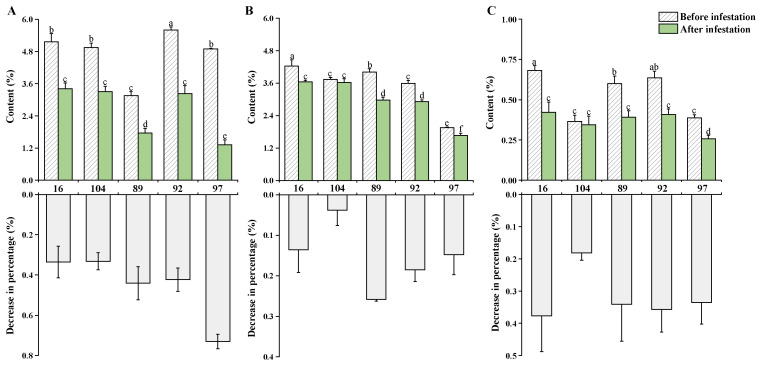
The content and change of (**A**) soluble sugar; (**B**) sucrose; and (**C**) fructose before and after aphid infestation treatment. Each value was the mean (±SE) of three replicates. Tukey’s multiple range tests at *p* < 0.05 were used to compare means by SPSS. The letters (a−f) in the figures indicate a significant difference among experimental sugarcanes.

The food obtained by aphids from the phloem flow is mainly composed of carbohydrates, mostly sucrose [25]. Therefore, generally sugars decline after pest infestation. The soluble sugar, sucrose and fructose content before and after aphid infestation were determined in this study. Five days after infestation, the soluble sugar content of infested leaves showed a significant decrease compared to the uninfected plants (*p* = 0.05). The percentage of decrease in the soluble sugar content of the transgenic lines was obviously lower than that of the control lines (Figure 7A). The sucrose content of leaves also dropped, but the transgenic sugarcane did not show a significant level of decrease in sucrose content, especially PPA−104 (Figure 7B). The fructose content of the leaves also showed a decreasing trend five days after infestation. The fructose reduction of 37% in PPA−16 was basically the same as that of the control, and the reduction of 18% in PPA−104 was significantly lower than the other sugarcane lines (Figure 7C). Transgenic sugarcane PPA−104 exhibited the least reduction of all the sugars tested. The *PPA* transgenic sugarcane lines showed no significant differences in sugar content compared with the control lines before infestation, and were basically higher in sugar content after infestation. This indicates that the overexpression of *PPA* had no significant effect on the sugar content of the sugarcane leaves without pest infestation and was successful in reducing the pest−associated sugar decline effect.

### 2.6. Variation in Tannin and Chlorophyll Content after Aphid Infestation

**Figure 8:**
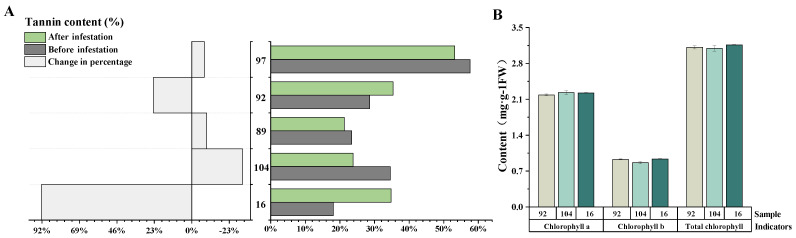
Tannin and chlorophyll content. (**A**) Determination of tannin content in sugarcane leaves before and after infestation treatment, three replicates for each sample. The percentage change was greater than 0 if tannin content increased after infestation, and lower than 0 if it decreased. (**B**) The mean chlorophyll content of the positive first leaf of *PPA* transgenic sugarcane lines (PPA−104, PPA−16) and control sugarcane line (92) at maturity, each in three replicates.

Tannin has been reported to function as pest resistance [23]. In our study, no significant differences in tannin content were detected between the transgenic sugarcane lines and the control lines before infestation treatment, as well as after treatment (Figure 8A). However, there was a significant increase in tannin in sugarcane PPA−16 after the infestation, with the tannin content almost doubling. This may be a result of the highest *PPA* expression being in PPA−16 and needs further exploration. Furthermore, the determination of chlorophylls showed no significant variance between the transgenic sugarcane lines and the control lines either before or after treatment (Figure 8B).

## 3. Discussion

Highly aphid−resistant sugarcane lines were generated by overexpressing the phytolectin *PPA* gene and were confirmed by an insect infestation treatment. It was evidenced by the experiments that five days after infestation treatment, the number of aphids surviving on the leaves of *PPA* transgenic sugarcane lines were obviously lower than that of the control line. Moreover, PPA−16, having the highest *PPA* gene expression, exhibited the highest aphid resistance, indicating that there was a positive correlation relationship between aphid resistance and the expression level of the *PPA* gene. The results of this study were consistent with the results of *PPA* transformation in tobacco and wheat [18,19].

The current hypothesis regarding the anti−insect mechanism of phytolectin is that phytolectin can specifically bind to glycoproteins located in the digestive tract of insects, which could reduce the permeability of the membrane and impede the transport and absorption of substances, directly leading to insect death [26]. However, the deep mechanisms of phytolectin−associated aphid resistance have not yet been fully demonstrated so far, and the current hypothesis is not supported by direct evidence from studies. The results of this study confirm that sugarcane plants with small and dense leaf stomata have better defense against aphids, and that the stabilization of the antioxidant enzyme system, sugar and tannin content of sugarcane after aphid infestation is important for preventing further infestation.

Stomata patterning differences were reportedly found in bred sugarcane cultivars that were identified as aphid resistant [22]. The stomata on the back of leaves are a natural defense for plants, and stomatal size and density can indicate a plant’s capability of resistance to insects and pathogens to some extent [27]. We hence observed the stomata, and found that the change of stomata patterning due to *PPA* overexpression was exactly the same as that of the description of other aphid−resistant sugarcane hybrid cultivars: the stomata were more abundant in number and reduced in size. We hypothesized that *PPA* could affect stomata development through some pathways, and that stomata patterning, which is associated with plant transpiration, may further influence aphid passive feeding. This idea was supported by a report that aphids can cause the closure of stomata in affected leaves, which can help the plant keep osmotic pressure for aphids to suck up water passively [24].

To further analyze the *PPA*−associated resistance mechanism, we assayed the content of main physiological indicators, including antioxidant enzymes, proteins, sugar, tannin and chlorophylls. In plants, reactive oxygen species (ROS) can be produced by normal physiological metabolic reactions. The synergistic effect of superoxide dismutase (SOD) and peroxidase (POD) can effectively inhibit the damage of ROS to the plant [28,29], as well as ascorbate peroxidase (APX) [30]. Insect stress can easily lead to ROS accumulation; however, antioxidant enzymes usually decrease after insect infestation, which is probably due to insect induction, for insects to better feed on plants after long−term co−evolution. Therefore, the capability of plants to keep antioxidant enzyme activities stable or even to enhance their activities is found to be related to a resistance to insect stress [31,32]. In our study, a comparison of antioxidant enzymes between transgenic sugarcane lines and control lines revealed that the expression of the *PPA* gene led to a decreased reduction of antioxidant enzymes, with some transgenic lines even increasing in APX and POD content. This indicated that PPA was related to antioxidant systems, the mechanism of which needs further investigation.

Aphids make use of plant sap to absorb sugars and produce honeydew. Aphids store large amounts of sucrose in their tissues after consuming plant sap, which may originate directly from the plant or possibly be converted from ingested fructose and glucose. It was reported that the Pea aphid *Acyrthosiphon pisum* can consume more than 40% of sucrose from artificial feed into the aphid tissues [33]. However, another study reported that despite the absence of sucrose in some carrot seedlings, fructose, sucrose and eight other sugars were found to have substantially increased in the honeydew of *Myzus persicae* after parasitizing carrot plantlets [34]. Therefore, aphids can consume a large variety of sugars to change for their own use. In our study, we measured the content of different sugar sources before and after aphid feeding. The transgenic sugarcane line PPA−104 exhibited the least reduction of all sugars, followed by PPA−16, which was conducive to maintaining sugar stability and providing a more stable internal environment to resist aphids. No significant difference in sugar content between *PPA* overexpression lines and control lines was detected before infestation, indicating that *PPA* did not affect normal sugar accumulation without insect infestation.

Tannin is an important polyphenolic polymer in plants and has anti−nutritional properties. Not only does its bitter taste reduce the appetite of animals, it also inhibits the activity of rumen digestion and microbial enzymes, which weakens the absorption of nutrients [23]. Plants with a high tannin content are more resistant to insects than those with a low tannin content. As confirmed by this study, a substantial increase in tannin in PPA−16 after infestation was found, with PPA−16 exhibiting the highest *PPA* expression. This high tannin content could prevent further aphid infestation, but it remains to be further investigated whether tannin plays a decisive role in the cause of aphid mortality. Other than that, chlorophylls were not identified as having been changed in either the *PPA* overexpression lines or control lines.

In this study, we generated *PPA*−mediated aphid−resistant sugarcane transgenic lines and identified one leader line that could be used for future sugarcane breeding. The breeding of wooly aphid−resistant transgenic sugarcane is not only important for environmental protection and sustainable agricultural development, but also provides insights for subsequent in−depth research on plant lectin−related insect resistance mechanisms and crop pest prevention and control.

## 4. Materials and Methods

### 4.1. Plant Materials and Ceratovacuna lanigera Zehntner Sampling

The transformation of the material sugarcane variety Zhongzhe 1(ZZ1) was bred by the sugarcane team at Guangxi University and planted in the Guangxi University experimental field (Nanning, China). The tops of sugarcane were used for tissue culture. The *PPA* gene was synthesized.

Ceratovacuna lanigera Zehntner used in this study was sampled at the New City of Agricultural Science of Guangxi University (Fusui, China) and was then raised in the experimental greenhouse of Guangxi University.

### 4.2. Transformation of the PPA Gene in Sugarcane

Overexpression vector pCAMBIA2301−*PPA* for plants was constructed through the method of double digestion and followed by T4 ligase ligation. Induction of sugarcane direct embryos, biolistic transformation, regeneration and selection of resistant materials were referred to previous studies [35,36]. Sugarcane tops of ZZ1 were sliced into 1–2 mm slices under aseptic conditions and moved onto a direct embryogenesis medium (DEM). The explant tissue was cultured at 28 °C and under a light intensity of 30 µmol m^−2^ s^−1^ intensity with 16:8 h (light/dark) photoperiod. Transformation was performed after about 13 days of tissue culture with one−time subculture. The plasmids were mixed with gold particles and bombarded into explant cells by a Biolistic PDS−1000/He apparatus. After that, the sugarcane embryonic tissues were recovered for four days before selection on a medium containing 30 mg/L geneticin (G418). G418−resistant sugarcane plantlets were rooted and transplanted into soil and grown in a climate chamber. Genomic DNA was extracted from the leaves to verify successfully transformed sugarcanes.

### 4.3. Expression Analysis of the PPA Gene

Total RNA was extracted from the positive fourth leaf by a Total RNA Extractor Kit (Sangon Biotech, Shanghai, China). The cDNA was obtained by PrimeScript™ RT reagent Kit with gDNA Eraser (Takara Branch, Beijing, China). A pair of specific primers were designed for the *PPA* gene and the *GAPDH* gene was used as an internal reference gene (Supplementary Table S1) [37]. Real−time quantitative PCR was conducted to identify the expression level of the *PPA* gene in the transgenic sugarcane plants with TB Green^®^ Premix Ex Taq™ II (Takara Branch, Beijing, China). Three biological replicates and three technical replicates were designed for each sample. The PCR amplification was 95 °C for 30 s, 40 cycles of 95 °C for 5 s, 58 °C for 30 s, 95 °C for 5 s, 60 °C for 1 min, 95 °C for 1 s, and final extension was at 50 °C for 30 s. Relative quantitative analysis of experimental results data was calculated with the 2^−ΔΔCT^ method and three replications were performed.

### 4.4. Aphid Resistance Tests and Stomatal Microscope Observation on Sugarcane

The sugarcane woolly aphids were placed on the tested plants in the greenhouse of Guangxi University. The evenly distributed aphid leaves were divided into five parts. The leaves with aphids were gently tied with strings to the positive fourth leaves of the sugarcane, including transgenic sugarcane lines and control lines. We recorded the temperature and humidity of the greenhouse every morning, midday and evening to avoid environmental damage to the aphids. The number and activity of aphids on the leaves were observed on a daily basis. After sweeping out the aphids and recovering for three days, two aphid inoculations were conducted. For microscopy experiments, leaves approximately 3 cm long were quickly placed in fixative (4 °C) and each sample was repeated at least three times. The film was prepared via nail polish print method and later observed under a light microscope with a suitable magnification of 300 times magnification and an area of 1.285 mm^2^.

### 4.5. Antioxidant Enzyme and Total Protein Content Measurement

Fresh leaves (0.2 g) were homogenized under ice−cold conditions in a 3 mL 50 mM phosphate buffer (pH 7.8) containing 0.2 mM EDTA and 2% PVP. In addition, when measuring ascorbate peroxidase (APX), ASA was added to the buffer. After centrifugation at 12,000× *g* for 20 min, the supernatant was retained for measurement. Activity of ascorbate peroxidase (APX), catalase (CAT), peroxidase (POD) and superoxide dismutase (SOD) were measured with reference to the method before [38,39,40,41]. During the experiment, some modifications were applied. To measure the protein content, 3 mL of Thomas Brilliant Blue was added to 15 uL of supernatant and measure the absorbance value at 595 nm. The experiment was repeated three times (three different aphid−inoculated leaves were selected).

### 4.6. Determination of Fructose, Sucrose and Soluble Sugar Content

Initially, we put 0.05 g of dried leaves into 4 mL of 80% ethanol. Afterwards, the tubes were kept in an 80 °C water bath for 40 min with constant stirring, and then centrifuged for 6 min. The supernatant liquid was extracted and repeated once. We added 10 mg of activated carbon to the supernatant and put it into an 80 °C water bath for decolorization for 30 min, and then filtered them to obtain solution A for measurement. At the same time, a sucrose standard curve and fructose standard curve were made. To measure fructose, we took 2 mL of A above and added 2.8 mL of 10 mol/L HCl, and 0.8 mL of 0.1% resorcinol. After mixing, we placed them in an 80 °C water bath for 10 min and measured the absorbance value at 480 nm when cooling. Lastly, we calculated the content referencing standard curve. To measure sucrose, we took 2 mL of A above and added 150 uL of 2 mol/L NaOH. We placed them in an 80 °C water bath for 10 min and then added 2.1 mL of 10 mol/L HCl and 0.6 mL of 0.1% resorcinol until cooled to room temperature. We placed them in an 80 °C water bath for 10 min again after mixing well. When the solution was cooled, we measured the absorbance value at 480 nm and calculated the content referencing standard curve. The content of soluble sugars was determined by using the anthracene colorimetric method. Firstly, we took 0.5 g of leaves, put them in 25 mL of boiling water and boiled them in a water bath for 10 min. We filtered the leaves and set the volume to 50 mL, to get solution B. We obtained 2 mL of solution B in a pipette and diluted it to 50 mL, and repeated this once, adding 0.5 mL of anthrone and 5 mL of concentrated sulfuric acid. Lastly, we measured the absorbance value at 620 nm and calculated the content referencing standard curve. The experiments were repeated at least four times.

### 4.7. Measurement of Tannin and Chlorophyll

The Folin−Denis method was adopted to measure the tannin. The first step was to prepare Folin−Denis color developer, 50 g of sodium tungstate, 10 g of Phosphomolybdic acid hydrate, dissolved in 375 mL of distilled water, then 25 mL of 85% H3PO4, refluxed in a water bath for 2 h. After cooling, the volume was fixed and stored in a 500 mL brown bottle. Exactly 1 g of dried leaves was put into a 250 mL conical flask and extracted with 80% ethanol 3 times, for 2 h each time, and filtered to get solution A. Exactly 1 mL of solution A was put in a 25 mL volumetric flask, and 3.0 mL of Folin−Denis color developer was added, then 5.0 mL of 7% Na_2_CO_3_ solution. The volume was set to the scale with distilled water, mixed and keep in 60 degrees for 20 min, and absorbed with a 1 cm cuvette at a wavelength of 756 nm. The experiment was repeated three times. Chlorophyll content in leaves was determined by ethanol and acetone extraction [42].

## 5. Conclusions

Taken together, the results of our study determined that the transformation of the *PPA* gene effectively improved the resistance of sugarcane to aphids. In addition, the higher expression level of the *PPA* gene, the greater effect of aphid resistance. Preliminary investigation of the mechanism of *PPA*−associated aphid resistance was conducted and discussed. After aphid infestation, we found changes in different plant physiological characteristics caused by *PPA* expression, including smaller and denser leaf stomata and better stabilization of the antioxidant enzyme system, sugar and tannin content of sugarcane, all of which may affect the aphid resistance capability of sugarcane. These overall changes to plant physiological characteristics may be due to changes to some pathways triggered by *PPA* overexpression, which need to be further studied.

## Data Availability

Not applicable.

## Supplementary Materials

The following supporting information can be downloaded at: https://www.mdpi.com/article/10.3390/ijms23137195/s1.
