# Supplementary material for: Expression of Pinellia pedatisecta Agglutinin PPA Gene in Transgenic Sugarcane Led to Stomata Patterning Change and Resistance to Sugarcane Woolly Aphid, Ceratovacuna lanigera Zehntner
Source: Int J Mol Sci. 2022 Jun 28;23(13):7195. doi: 10.3390/ijms23137195 (PMC9266654; doi:10.3390/ijms23137195)
Supplement: Supplementary file 1 [file ijms-23-07195-s001.zip › ijms-1755254-supplementary.pdf]

Table S1. Primers for qPCR.

Primer name	Sequence（5'—3'）
PPA-F	ATGGCCTCCAAGCTCCTCC
PPA-R	GACCAAGTCGAAGTCGCCG
GAPDH-F	CACGGCCACTGGAAGCA
GAPDH-R	TCCTCAGGGTTCCTGATGCC

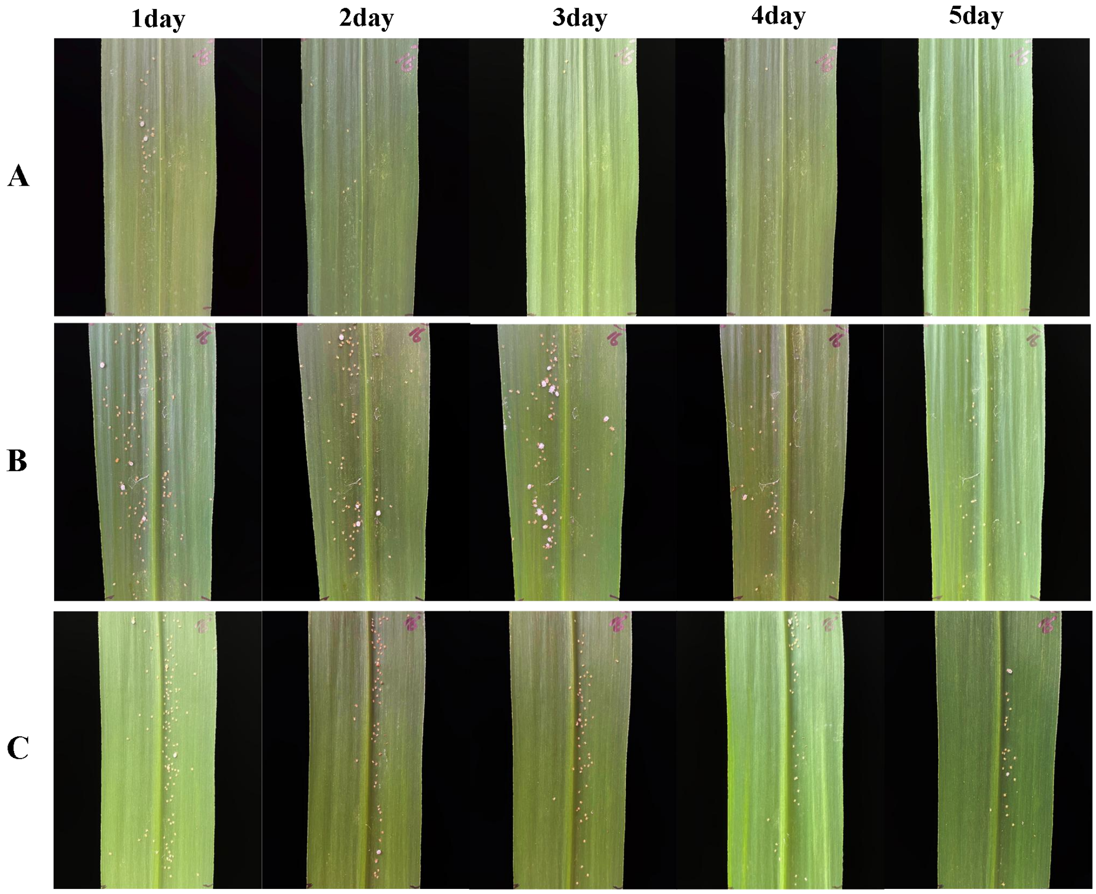

Figure S1. Aphids resistance tests on *PPA* transgenic sugarcane line 16. Record aphids number change five days after infestation, and (A) (B) (C) were three replicates respectively.

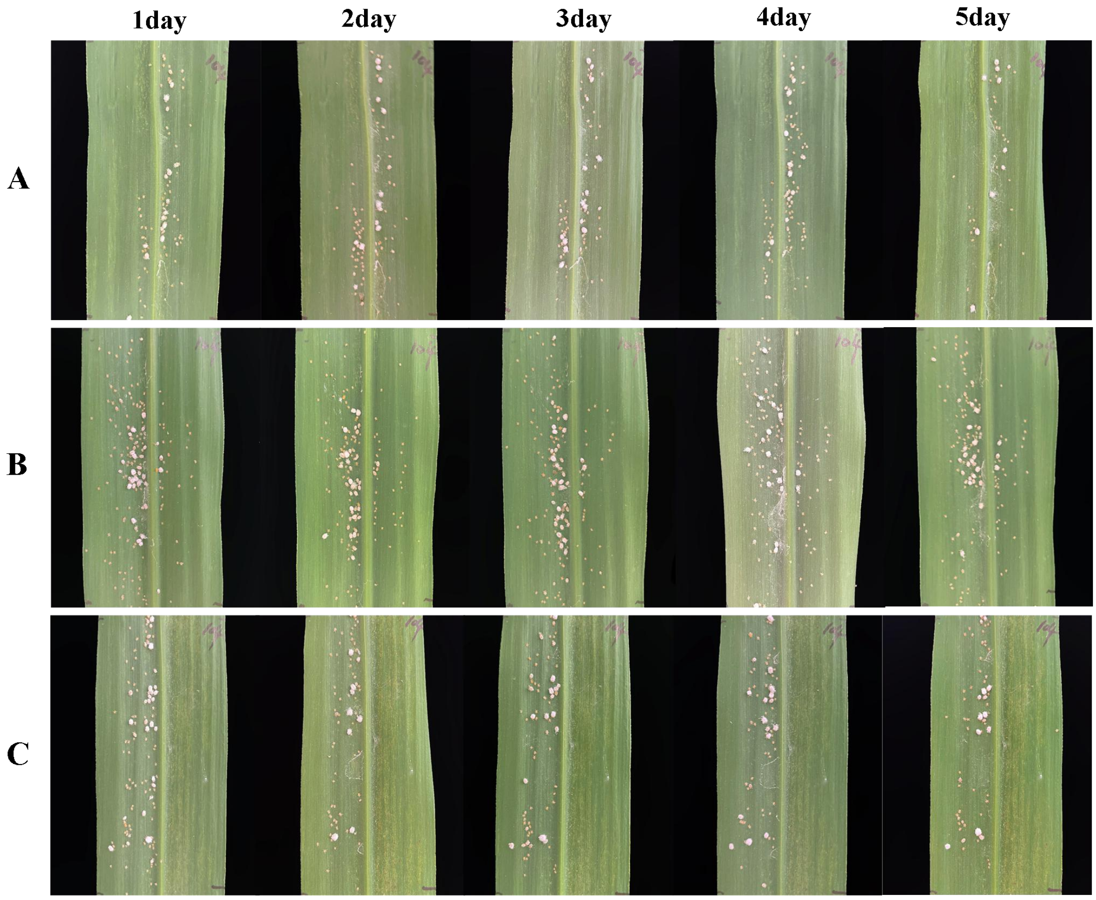

Figure S2. Aphids resistance tests on *PPA* transgenic sugarcane line 104. Record aphids number change five days after infestation, and (A) (B) (C) were three replicates respectively.

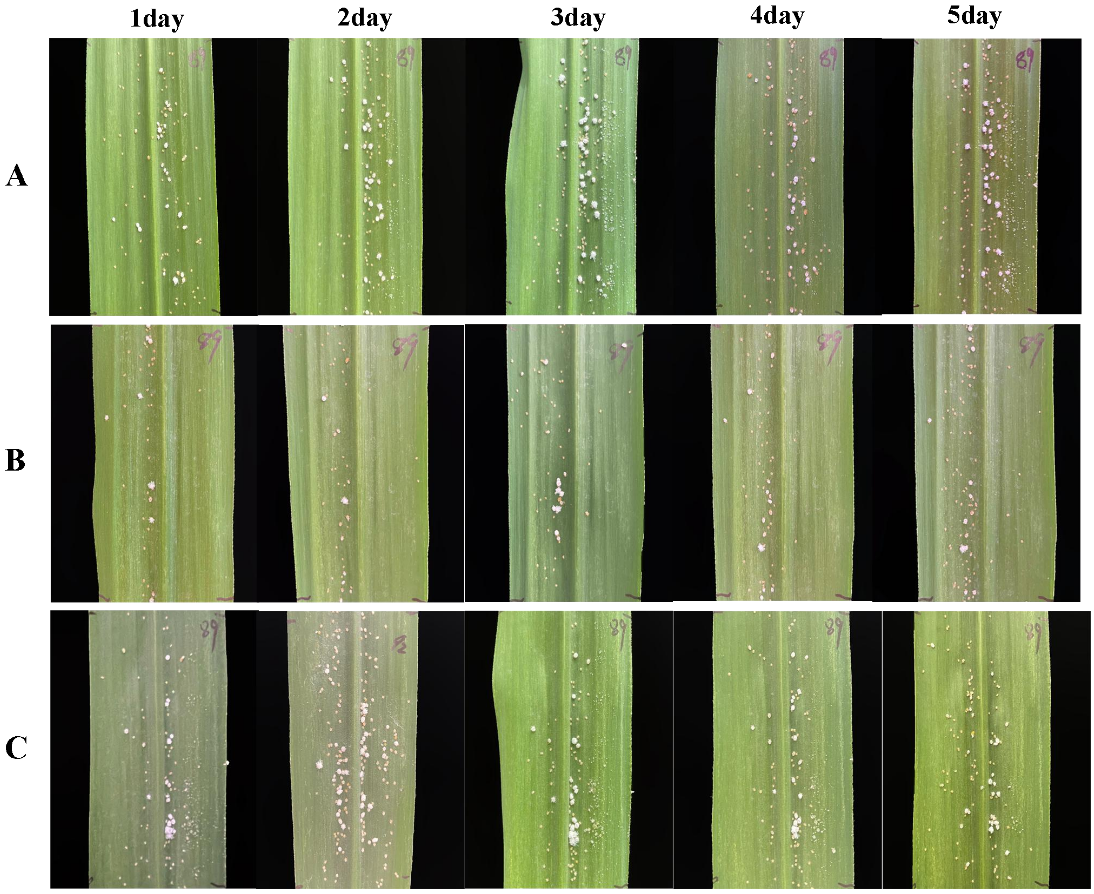

Figure S3. Aphids resistance tests on control transgenic sugarcane line 89. Record aphids number change five days after infestation, and (A) (B) (C) were three replicates respectively.

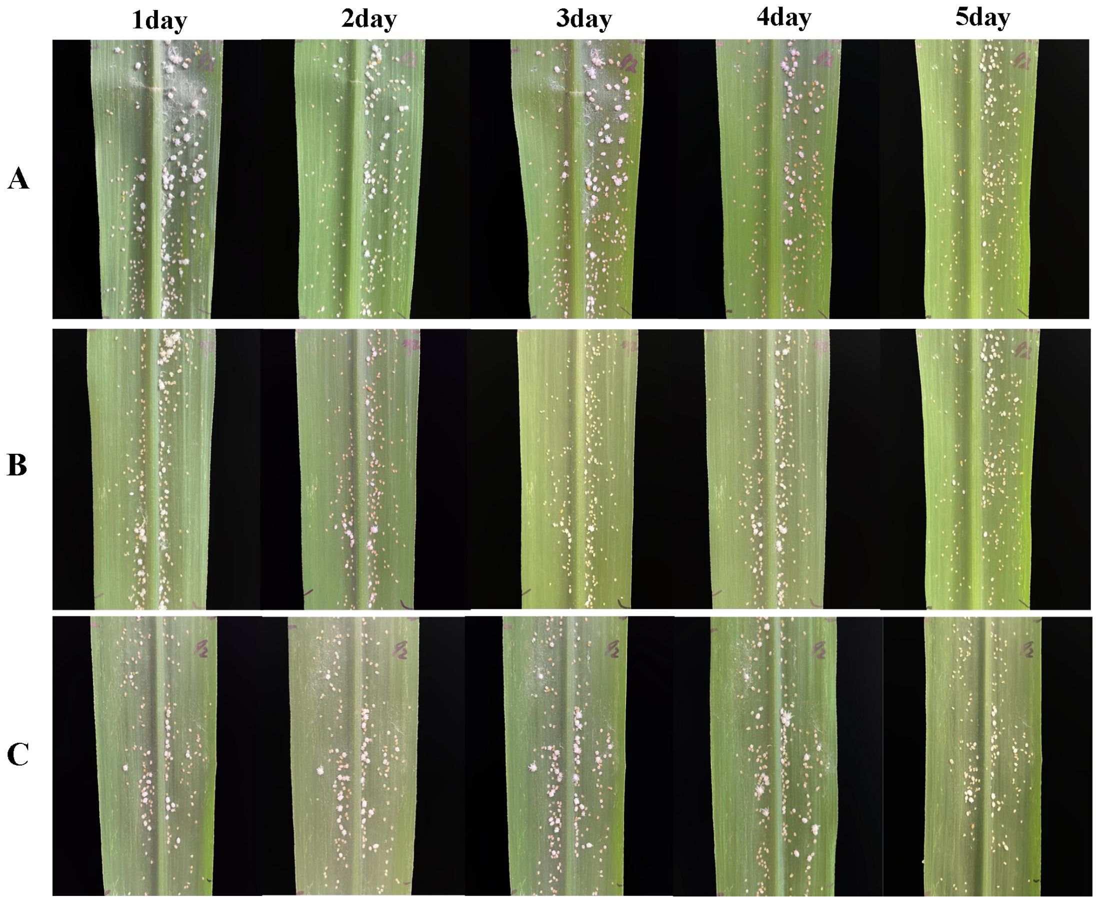

Figure S4. Aphids resistance tests on control transgenic sugarcane line 92. Record aphids number change five days after infestation, and (A) (B) (C) were three replicates respectively.

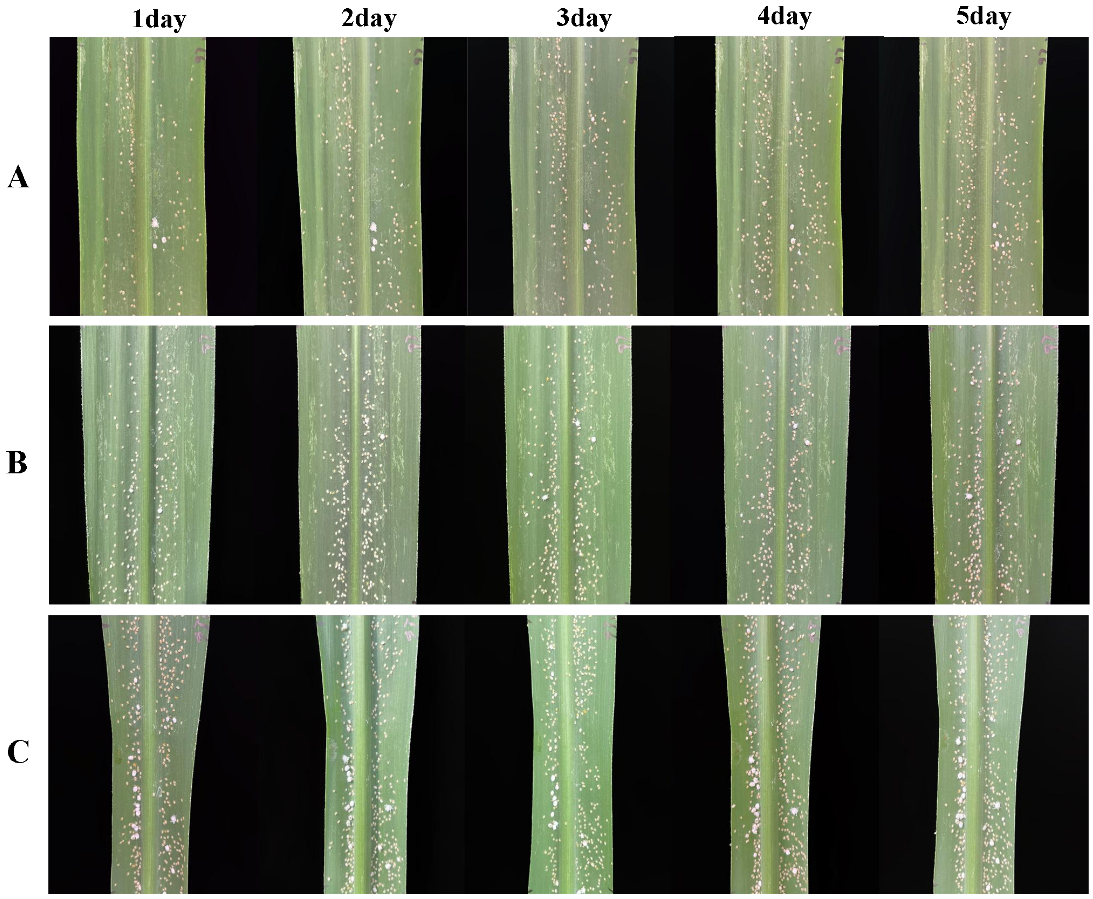

Figure S5. Aphids resistance tests on control transgenic sugarcane line 97. Record aphids number change five days after infestation, and (A) (B) (C) were three replicates respectively.
